# Genome-Wide Nucleosome Positioning Is Orchestrated by Genomic Regions Associated with DNase I Hypersensitivity in Rice

**DOI:** 10.1371/journal.pgen.1004378

**Published:** 2014-05-22

**Authors:** Yufeng Wu, Wenli Zhang, Jiming Jiang

**Affiliations:** Department of Horticulture, University of Wisconsin-Madison, Madison, Wisconsin, United States of America; University of Minnesota, United States of America

## Abstract

Nucleosome positioning dictates the DNA accessibility for regulatory proteins, and thus is critical for gene expression and regulation. It has been well documented that only a subset of nucleosomes are reproducibly positioned in eukaryotic genomes. The most prominent example of phased nucleosomes is the context of genes, where phased nucleosomes flank the transcriptional starts sites (TSSs). It is unclear, however, what factors determine nucleosome positioning in regions that are not close to genes. We mapped both nucleosome positioning and DNase I hypersensitive site (DHS) datasets across the rice genome. We discovered that DHSs located in a variety of contexts, both genic and intergenic, were flanked by strongly phased nucleosome arrays. Phased nucleosomes were also found to flank DHSs in the human genome. Our results suggest the barrier model may represent a general feature of nucleosome organization in eukaryote genomes. Specifically, regions bound with regulatory proteins, including intergenic regions, can serve as barriers that organize phased nucleosome arrays on both sides. Our results also suggest that rice DHSs often span a single, phased nucleosome, similar to the H2A.Z-containing nucleosomes observed in DHSs in the human genome.

## Author Summary

The fundamental unit of chromatin is the nucleosome, which consists of 147 bp of DNA wrapped around a histone octamer containing four core histones (H3, H4, H2A, and H2B). Nucleosome positioning in the genome affects the DNA accessibility for regulatory proteins, and thus is critical for gene expression and regulation. Genomic regions associated with regulatory proteins are associated with a pronounced sensitivity to DNase I digestion, and are thus called DNase I hypersensitive sites (DHSs). It is well known that only a subset of nucleosomes are reproducibly positioned in eukaryotic genomes. However, it is less clear what factors determine genome-wide nucleosome positioning, especially in intergenic regions. We mapped both nucleosome positioning and DHS datasets across the rice genome. We discovered that DHSs located in a variety of contexts, both genic and intergenic, were flanked by strongly phased nucleosome arrays. We confirmed the same association of DHSs with phased nucleosomes in the human genome. We conclude that genomic loci associated with a diverse set of regulatory proteins are major determinants of nucleosome phasing, and this is true in both genic and intergenic regions.

## Introduction

The fundamental unit of chromatin is the nucleosome, which consists of 147 bp of DNA wrapped around a histone octamer containing four core histones (H3, H4, H2A, and H2B) [1]. Since the DNA has to bend sharply around the surface of the histone octamer, flexible or intrinsically curved sequences are favorable for nucleosome formation [2]. In contrast, poly(dA:dT) stretches, which are intrinsically stiff, have been shown to be unfavorable for nucleosome formation and are more enriched in linker sequences [3]–[5]. The intrinsic properties of poly(dA:dT) are also important for nucleosome depeltion, promoter accessibility and transcriptional activity [6]. *In vitro* nucleosome assembly studies in yeast (*Saccharomyces cerevisiae*) and *Caenorhabditis elegans* have confirmed the DNA sequence preferences in nucleosome formation [7], [8]. However, nucleosome organization *in vivo* is determined by several factors that can override the sequence preferences, including gene transcription, action of nucleosome remodeling complexes, and presence of histone variants and histone modifications [2], [6]. In fact, a sequence preference-based model could only explain ∼50% of the *in vivo* nucleosome positions in *S. cerevisiae* [9]. Similarly, only 20% of the human genome is occupied by preferentially positioned nucleosomes [5]. It is important to take such numbers with caution, however, as the calculations are affected by the sequencing methodology and the cell/tissue types used in analysis [10].

Relationships between nucleosome organization and gene expression have been well demonstrated in several model eukaryotes. Phased nucleosome arrays have been observed on both sides of the promoters of active genes [5], [8], [11]–[15]. The promoter itself was traditionally considered to be nucleosome free or depleted, producing what is often called a “nucleosome-free region” (NFR). The first nucleosome downstream and upstream of the promoter are named +1 and −1 nucleosomes, respectively. Nucleosomes after the +1 or before the −1 nucleosome become progressively less phased. Nucleosome positioning in the human genome appears to correlate with the levels of Pol II in the promoter region: better phasing is observed with higher levels of Pol II and less phasing with lower levels of Pol II [13]. So far, the majority of the nucleosome organization studies have been focused on genomic regions associated with transcription. It is unclear, however, what factors determine nucleosome positioning in intergenic regions.

Rice (*Oryza sativa*) has been used as model species for plant genome research. The rice genome is relatively small (∼400 Mb) and is one of the best sequenced genomes in higher eukaryotes [16]. Various genome-wide genomic and epigenomic datasets have been developed in rice [17]–[22]. Thus, rice provides an excellent model system for nucleosome positioning studies. We generated genome-wide nucleosome positioning data in rice. We mapped both nucleosome positioning and DNase I hypersensitive site (DHS) datasets in the rice genome. We discovered that DHSs associated with different genomic regions, including promoters, genes, and intergenic regions, were all flanked by strongly phased nucleosome arrays. Our results support the barrier model for nucleosome organization. The DHSs, which are likely bound to regulatory proteins, can serve as the barriers to organize phased nucleosome arrays on both sides. Thus, genome-wide nucleosome positioning appears to be orchestrated by genomic regions associated with regulatory proteins.

## Results

### Rice DHSs were flanked by phased nucleosomes

DHSs are markers of regulatory DNA and span all classes of *cis*-regulatory elements, including promoters, enhancers, insulators, silencers and locus control regions [23]. We applied a strategy of mapping both nucleosome positioning and DHS datasets to examine whether nucleosome positioning is associated with all *cis*-regulatory elements across the rice genome. All datasets used in the analysis were developed using rice leaf tissue at the same developmental stage (see Materials and Methods). Rice chromatin was digested by micrococcal nuclease (MNase) into mono-nucleosome size. Mono-nucleosomal DNA was isolated and sequenced (MNase-seq) using Illumina sequencing platforms. We obtained a total of 38 million (M) single-end reads from our first MNase-seq experiment and mapped ∼26 M to unique positions in the rice genome. We also conducted pair-end sequencing of an independent MNase-seq library, obtained 274 M paired-end reads, and mapped ∼231 M read pairs to unique positions in the rice genome.

**Figure 1:**
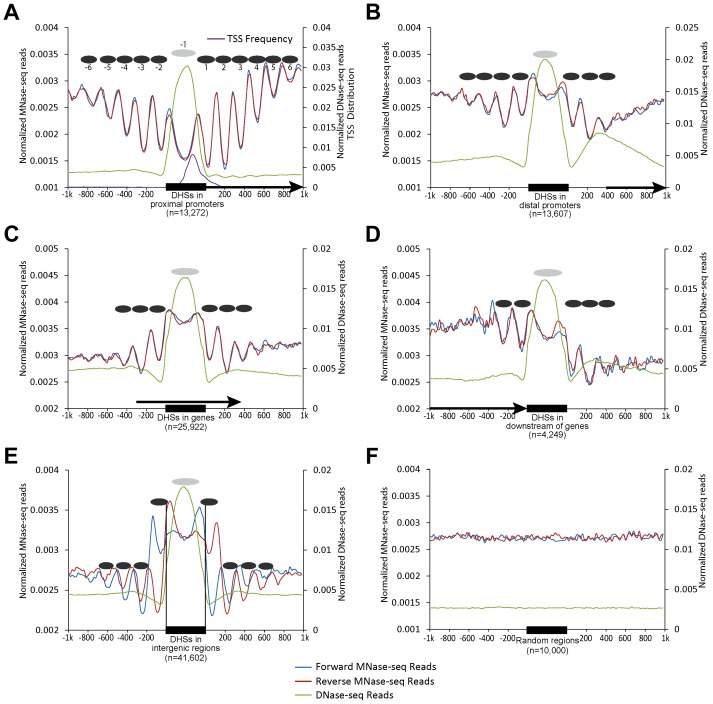
Patterns of nucleosome positioning around DHSs in the rice genome. The nucleosome positioning profiles were shown around the DHSs located in (A) proximal promoters (within 200 bp upstream of a TSS); (B) distal promoters (200–1000 bp upstream of a TSS); (C) within genes; (D) downstream regions of genes (within 200 bp downstream of gene transcription); (E) intergenic regions and (F) 10,000 randomly selected genomic regions. Y-axes show normalized reads (read number in per bp genome in per million reads) within 1 kb upstream and downstream around the DHSs. Ellipses indicate the nucleosomes within (grey) and outside (black) of DHSs. Arrows in (A–D) indicate the direction of gene transcription. Single-end MNase-seq reads were used in mapping nucleosome positioning.

We previously identified a total of 97,975 DHSs (leaf tissue) in the rice genome [24]. We grouped these DHSs into five categories based on their locations in the genome: 13,272 in proximal promoters (within 200 bp upstream of a TSS), 13,607 in distal promoters (200–1000 bp upstream of a TSS), 25,922 within genes, 4,249 in downstream regions of genes (within 200 bp downstream of the end of transcription), and the remaining 41,602 in intergenic regions. We then aligned both DNase-seq and MNase-seq reads to the rice genome. Strikingly, we observed peaks of read alignments oscillating from both sides of DHSs, indicating the presence of regularly spaced, phased nucleosomes. This phenomenon was evident both in forward and reverse oriented reads (represented by positions of their 5' ends) and in both single-end reads (Figure 1) and paired-end reads (Figure S1). The highest amplitudes of the oscillations were immediately adjacent to boundaries of the DHSs, suggesting that the nucleosomes close to the DHSs were more phased than those far from the DHSs. Phased nucleosomes were not observed in regions flanking randomly selected genomic regions (Figure 1F). The pattern of phased nucleosome arrays surrounding the DHSs is highly similar to the phased nucleosomes surrounding the promoters of active genes reported in model animal species [5], [11], [13].

### Phased nucleosomes flanked both sides of transcription start sites (TSSs)

**Figure 2:**
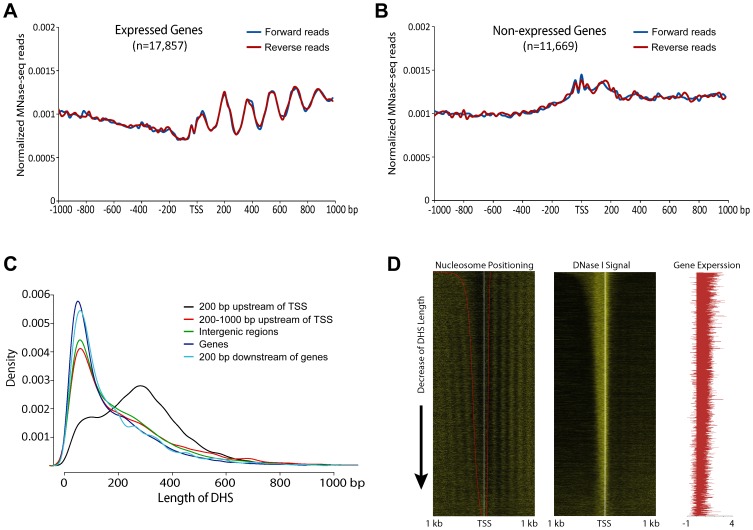
Phased nucleosome arrays flanked TSSs of rice genes. (A) Nucleosome positioning profile associated with active genes. Phased nucleosome arrays are detectable after the TSSs. (B) Nucleosome positioning profile associated with non-expressed genes. Phased nucleosome arrays are detected on either side of the TSSs. (C) Distribution of DHS length for five different DHS categories. Note: the length of DHSs associated with proximal promoters (black line) are more variable than the lengths of other DHSs. (D) Heatmap of nucleosome positioning associated with active genes. Left panel: All expressed genes were sorted by the length of DHSs located in proximal promoters. The 5′ ends of the MNase-seq reads were mapped within 1 kb upstream and 1 kb downstream of the TSS of each gene to show the boundaries of nucleosomes core and linker. The red line on the left heatmap indicates the boundaries of DHSs. With the same order of the genes as in the left panel, the 5′ ends of DNase-seq reads (middle panel) and the fragments per kilobase of exon per million fragments mapped (FPKM) value log10 transformation (right panel) were mapped to show the DNase I sensitivity and the expression level of each gene, respectively.

We also examined nucleosome phasing surrounding TSSs in the rice genome independently of DHSs. Clearly-phased nucleosomes were detected downstream of TSSs of expressed genes (Figure 2A), but not downstream of TSSs of non-expressed genes (Figure 2B), similar to the patterns observed in human and yeast genomes [5], [11], [13]. However, phased nucleosomes were not detected upstream of TSSs of expressed genes (Figure 2A), although phased nucleosomes were detected on both sides of the promoter DHSs (Figures 1A, 1B). In contrast, phased nucleosomes were observed on both sides of TSSs in human and yeast genomes [5], [11], [13].

We noticed that the average lengths of most DHSs in different genomic regions, except for those located in proximal promoters, were similar in the rice genome, with ∼50% DHSs in the size of 35–150 bp. In contrast, the lengths of DHSs in proximal promoters were more variable, including ∼79% DHSs >150 bp (Figure 2C). We suspected that the variable lengths of the DHSs in proximal promoters may mask the detection of nucleosome phasing in front of TSSs. We sorted the DHSs in proximal promoters based on lengths and examined the nucleosome positioning of all active genes associated with these DHSs. Phased nucleosomes were observed on both upstream and downstream of the TSSs of these genes (Figure 2D), which confirmed our prediction.

### Phased nucleosomes associated with IPA1-binding sites

**Figure 3:**
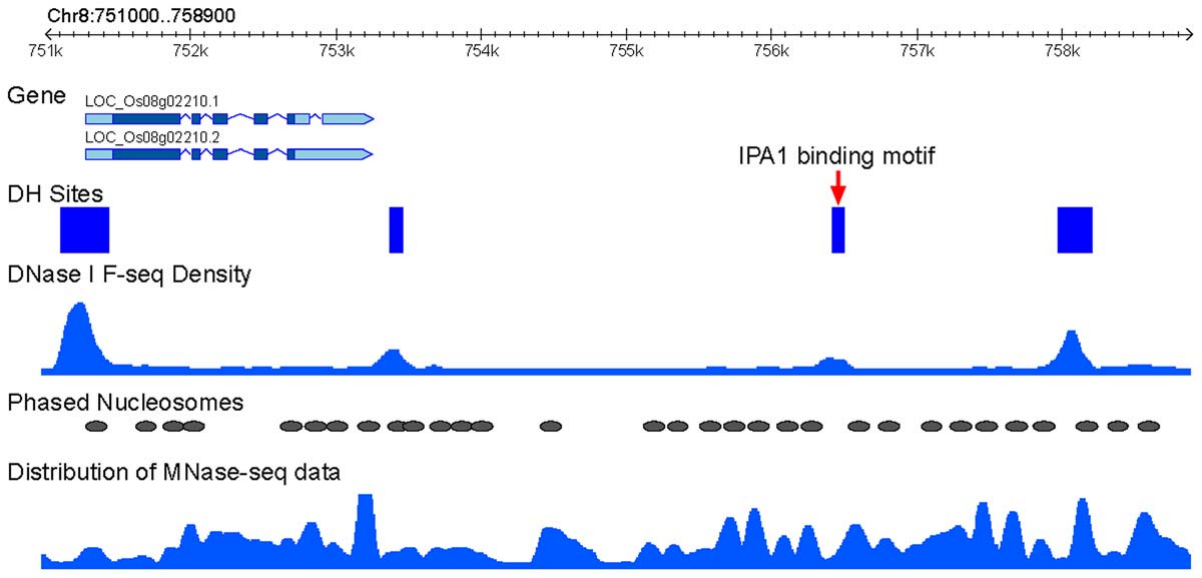
Association IPA1-binding sites with phased nucleosomes. An example of phased nucleosome arrays that flank an intergenic IPA1-binding site on rice chromosome 8. This binding site is overlapped with a DHS (red arrow). The distribution of MNase-seq data (dyad density calculated from paired-end reads by NucleR) and DNase-seq data (density calculated by F-seq) were used to present the nucleosome and DHS positions. Phased nucleosomes and DHS regions were also schematically marked.

We wanted to examine if phased nucleosomes are associated with the binding sites of specific rice transcription factors. IDEAL PLANT ARCHITECTURE1 (IPA1), a member of the SPL transcription factor family, is a key regulator in determining plant architecture and enhancing grain yield in rice [25]. A genome-wide IPA1-binding site map has recently been developed using ChIP-seq method and shoot apices tissue from 4-week-old rice seedling [26]. We found that 87.8% of the IPA1-binding sites (5,298 of 6,032) are associated with DHSs, despite of the fact that the DHS data was developed from 2-week-old seedling tissue [24]. An IPA1-binding site was considered to be flanked by phased nucleosome if the ±50 bp regions of the site overlap with a phased nucleosome. Under this criteria, 33.2% (1,757 of 5,298) of the IPA1-binding sites were flanked by phased nucleosomes (see an example in Figure 3), which is significantly higher than the frequency observed from 5,298 randomly selected regions (24.3%, binomial test, *p*<0.001). In addition, 5,197 and 2,898 of the IPA1-binding sites contain the IPA1-binding motif, GTAC, and another over-represented motif, TGGGC[C/T], respectively [26]. We found that 33.1% of the GTAC-containing sites and 36.2% of the TGGGC[C/T]-containing sites were flanked by phased nucleosomes under the same criteria.

### A predicted nucleosome spanned by rice DHSs

Mapping of both DNase-seq and MNase-seq datasets revealed peaked MNase-seq reads from both forward and reverse strands on both sides of DHSs (Figures 1A–1D). These results suggest that the DHS regions, although highly sensitive to DNase I cleavage, may span a structure that is more inhibitory to MNase digestion than the DHS-flanking regions. The most likely candidate for this predicted structure is a phased nucleosome within each DHS. This predicted nucleosome partially overlapped with the TSSs in proximal promoters (Figure 1A). We named this predicted nucleosome as “-1 nucleosome” because of its location in front of the TSS. The mapping results and our prediction are in agreement with a recent report that active promoters and other regulatory regions in the human genome are not nucleosome free, but are enriched with special nucleosomes containing both of the widely conserved histone variants H3.3 and H2A.Z [27]. These regions were previously considered as “nucleosome free” because nucleosomes carrying both H3.3 and H2A.Z are unusually unstable under the conditions that were commonly used for nucleosome preparation [27], [28]. This instability is believed to facilitate the access of transcription factors and regulatory proteins [27]. Nucleosome formation in promoters was detected during the activation of the zygotic genome of zebrafish [29].

The DHSs in intergenic regions were associated with a unique nucleosomal positioning pattern. The intergenic DHSs lacked the forward MNase-seq peak and the reverse MNase-seq peak, respectively, on the two sides of the DHSs (Figure 1E), suggesting that either these DHSs lack nucleosomes or the nucleosomes are poorly phased. Thus, intergenic DHSs are likely more dynamic with nucleosome occupation, which could mask the identification of a positioned nucleosome. Intergenic DHSs are highly enriched with enhancers in mammalian species [23], [30]. Thus, many of these regions may be associated with regulatory proteins in a cell type-specific manner, which would also mask the identification of positioned nucleosomes in datasets generated from tissues with mixed cell types, such as leaf. We previously demonstrated that rice DHSs generally lack histone modification marks associated with histone H3. However, intergenic DHSs were uniquely enriched with H3K27me3, suggesting a dynamic nucleosome occupation in these regions [24].

### Positioning of the -1 nucleosome relative to DHSs with different lengths in proximal promoters

**Figure 4:**
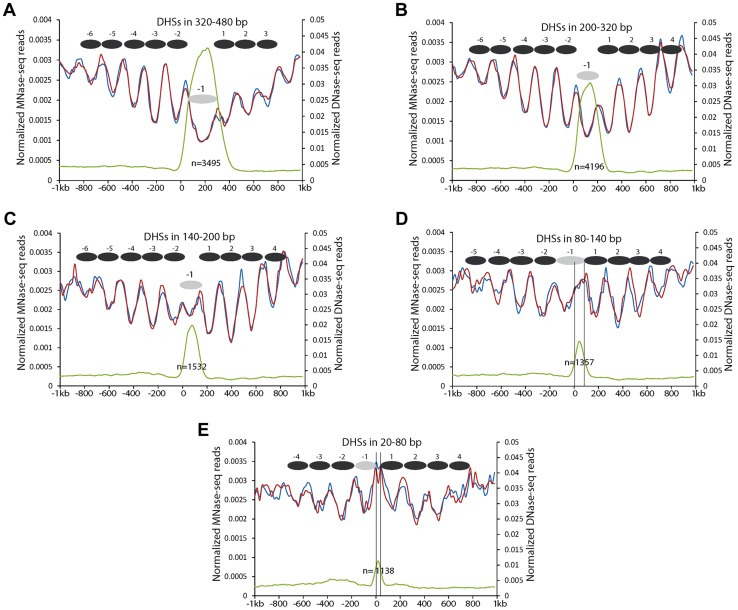
Nucleosome positioning profiles associated with DHSs with different lengths in proximal promoters. (A) DHSs in 320–480 bp. (B) DHSs in 200–320 bp. (C) DHSs in 140–200 bp. (D) DHSs in 80–140 bp. (E) DHSs in 20–80 bp. Y-axes show normalized reads of DNase-seq and MNase-seq. Zero on the X-axis indicates the boundary of DHSs toward short arm of the chromosomes. Black ellipses indicate the inferred nucleosomes. Grey ellipses indicate -1 nucleosomes within DHSs. Black vertical lines in (d, e) indicate the left and right boundaries of the DHSs inferred by DNase-seq reads.

Since the DHSs in proximal promoters were more variable in lengths (Figure 2C), we further investigated the positions of the -1 nucleosomes relative to the DHSs with different lengths. We divided the DHSs into five different groups based on their lengths (320–480 bp, 200–320 bp, 140–200 bp, 80–140 bp, and 20–80 bp, respectively). DHSs within the same group were aligned by their 5' ends. All DHSs with a length >140 bp showed a similar nucleosomal positioning pattern (Figures 4A, 4B, 4C). These DHSs appeared to span a single, phased nucleosome, although the DNA length of the DHSs in 320–480 bp is close to two nucleosomes, which may reflect nucleosomes with longer linkers, or nucleosomes tightly associated with other regulatory proteins. These results indicate that the -1 nucleosome in these promoters can accommodate variable amounts of DNA, perhaps reflecting the existence of diverse proteins that interact tightly with the -1 nucleosome or with promoter DNA.

The sizes of 2,495 DHSs (out of 11,718) in proximal promoters were <140 bp, which is shorter than the sequences required to wrap a single nucleosome. These DHSs did not appear to span a nucleosome, but appeared to be enriched in the 3′ portion of the -1 nucleosome (Figure 4D) or were located between the -1 and +1 nucleosome (Figure 4E). Thus, the small DHSs tend to be located in the linker regions. The levels of DNase I sensitivity within these small DHSs were clearly lower than those of the DHSs >140 bp (Figure 4).

### Longer linker between phased nucleosomes in intergenic regions

**Figure 5:**
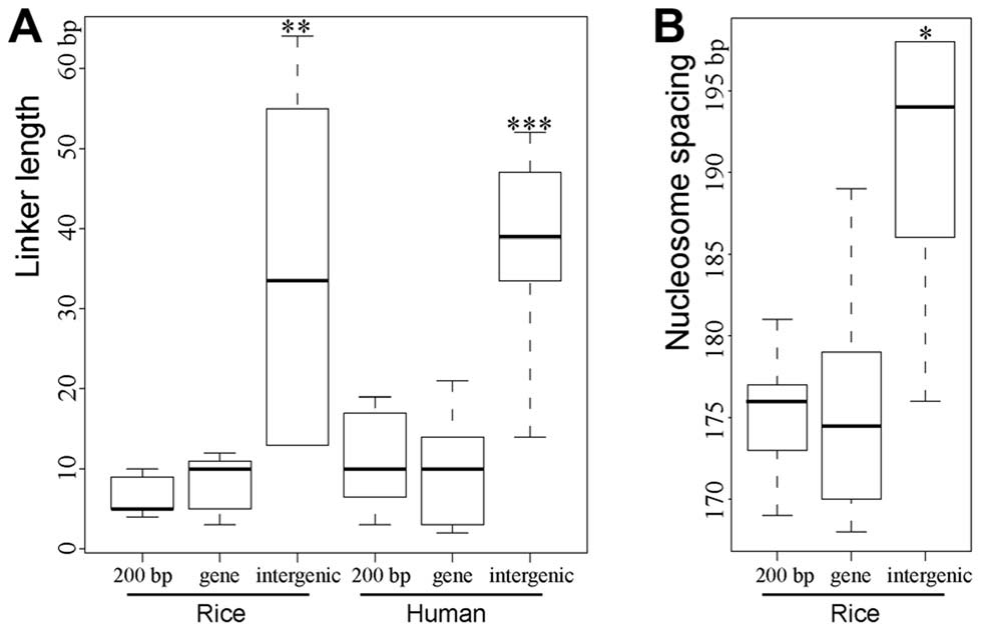
Boxplots of estimated lengths of linkers (A) and spacing (B) between the phased nucleosomes mapped close to DHSs. "***","**","*" indicated *p*<0.001, *p*<0.01, *p*<0.05, respectively, for the comparison of linker length/spacing between intergenic region and either regions within genes (“gene”) or in proximal promoters (“200 bp”).

We observed a superposition between the forward and reverse MNase-seq reads in genic and promoter regions, which indicates very little or no space between 5' ends of forward and reverse oriented reads (Figures 1A–1C). However, a clear shift between the forward and reverse reads was observed in intergenic regions (Figure 1E). We wondered if this shift was caused by longer linkers that connect the phased intergenic nucleosomes (Figure S2). We investigated the lengths of linkers between phased nucleosomes associated with different genomic regions. We used paired MNase-seq reads and employed 1-bp resolution to calculate the distribution of forward and reverse MNase-seq reads rather than using the 20-bp windows that we used for the other analyses. We measured the distance between maxima of adjacent peaks from reverse to forward strand, respectively, to estimate the length of the linkers between two adjacent nucleosomes. Assuming a constant nucleosome core DNA length of 147 bp, the average length of linkers between two phased nucleosomes in intergenic regions was 35.3 bp, which was significantly longer than the average lengths of linkers between two adjacent nucleosomes within genes (8.1 bp) and in proximal promoters (8.5 bp) (Figure 5A, *p*<0.005, Kolmogorov–Smirnov test). We also calculated linker lengths in the human genome using human MNase-seq data [13], and found a similar pattern as in rice: the linker length in intergenic regions in the human genome was 38.7 bp, compared to only ∼11.5 bp and 10.1 bp, respectively, for the linkers in proximal promoters and genic regions (Figure 5A).

A weakness of the above method of calculating linker length is that it is influenced by the severity of MNase digestion as MNase can either digest into the nucleosome core DNA or fail to completely digest the linker DNA. Thus, we used an alternative method to estimate the linker lengths in different genomic regions in rice. Since the position of the nucleosome center (dyad), which can be identified as the middle position of each paired-end read, is not affected by different levels of MNase digestion, we can calculate the spacing of between two adjacent nucleosomes using the midway point between paired MNase-seq reads rather than 5' ends. We found that the average spacing between two nucleosomes adjacent to intergenic DHSs was ∼191 bp (Figure 5B), which is significantly longer than the spacing between nucleosomes adjacent to DHSs in proximal promoters (175 bp) and genes (176 bp). The average spacing of nucleosomes associated with various histone modification marks was recently reported in human CD4+ T cells [5]. The average spacing of nucleosomes associated with H3K4me1 and H3K27ac, both euchromatin marks, are 178 bp and 179 bp, respectively. In contrast, the average spacing of nucleosomes associated with H3K9me3 and H3K27me3, both heterochromatin marks, are 205 bp [5]. Thus, linkers of nucleosomes in heterochromatin are significantly longer than the linkers of nucleosomes in euchromatin. These results are in agreement with the linker length difference in genic and intergenic regions observed in both rice and human genomes (Figure 5).

### Association of DHSs with phased nucleosomes in the human genome

**Figure 6:**
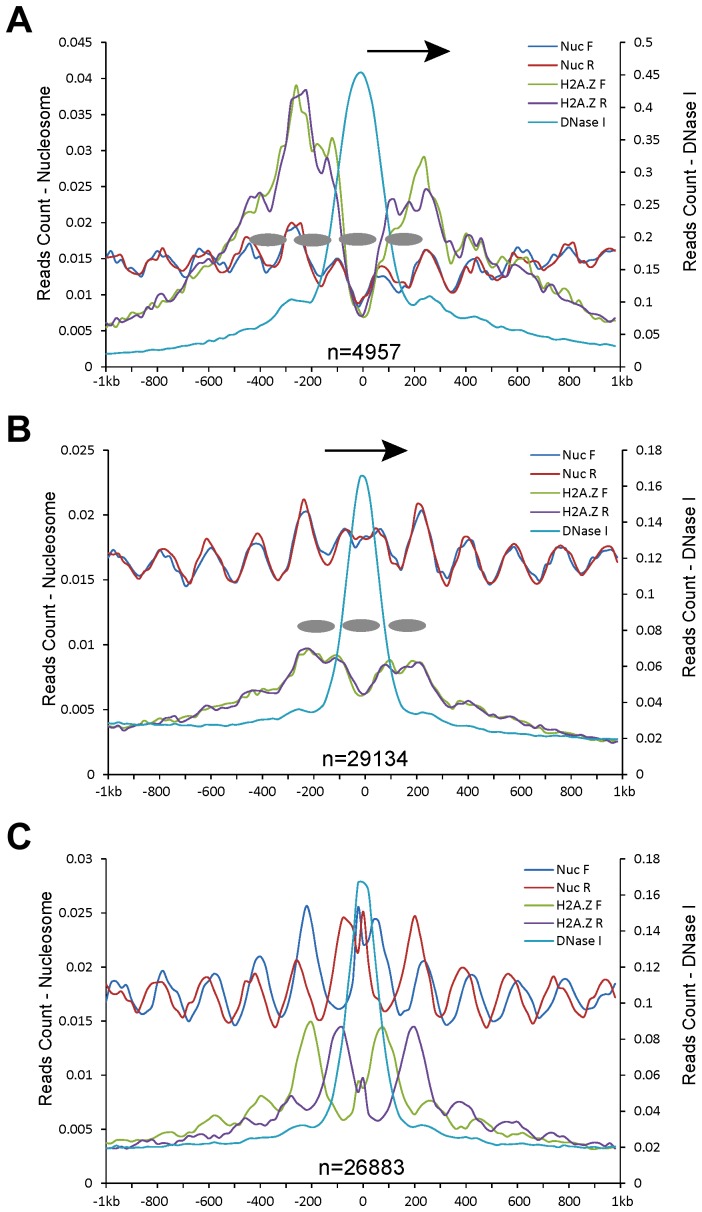
Patterns of nucleosome positioning around DHSs in the human genome. DHSs (data from CD4+ T cell line) were also divided into five different categories based on their genomic locations: **(A)** proximal promoters (within 200 bp upstream of a TSS); **(B)** within genes; and **(C)** intergenic regions. Y-axes show normalized MNase-seq reads (read number in per bp genome in per million reads). Zero on the x-axes indicates the most sensitive site of the aligned DHSs. Ellipses indicate phased nucleosomes with H2A.Z. Arrows in (A, B) indicate the direction of gene transcription.

We exploited the genomic datasets from the human genome to examine a similar association of DHSs with nucleosome positioning. Human CD4+ T cell line has been extensively used in epigenomics profiling, including histone modifications [31], nucleosome positioning [13], and DHS mapping [32]. We found that the relationship between DHSs and nucleosome positioning using datasets from the CD4+ T cell line was highly similar to the patterns observed in rice. The DHSs in proximal promoters (Figure 6A), genes (Figure 6B), and intergenic regions (Figure 6C) were flanked by phased nucleosomes. Interestingly, a similar shift between the forward and reverse MNase-seq reads was also observed in intergenic regions (Figure 6C).

Since H2A.Z-associated nucleosomes were found in regions that were previously thought to be nucleosome free, we investigated if DHSs in the human genome span H2A.Z-associated nucleosomes. Mapping of H2A.Z ChIP-seq dataset [31] together with DHS data [32] revealed a phased H2A.Z-associated nucleosome within DHSs in proximal promoters and genic regions in the human genome (Figures 6A, 6B). The intergenic DHSs tended to locate between two phased H2A.Z nucleosomes (Figure 6C). These results suggest that human DHSs span a phased H2A.Z nucleosome, which is also supported by previous data that a single H2A.Z nucleosome can be mapped within CTCF-binding sites in low-salt condition in the human genome [27]. The positions of the H2A.Z nucleosomes within human DHSs are highly similar to the implicated nucleosome within rice DHSs. Thus, we predict that the implicated nucleosome associated with rice DHSs likely contains H2A.Z, which serve as ‘place holders’ to facilitate binding of tanscription factors. The instability and dynamic replacement by regulatory proteins of these nucleosomes result in the DHSs in these genomic regions.

## Discussion

Genome-wide nucleosome positioning maps have been generated in several eukaryotes, including yeast [9], [11], [33]–[35], *Drosophila melanogaster* [12], *C. elegans* [36], humans [5], [10], [13], and *Arabidopsis thaliana* [37]. It has been well documented that only a subset of nucleosomes are phased in any genome. Most consistently, active genes form highly phased nucleosomes flanking the TSSs, which led to the suggestion that transcription may promote nucleosome organization [8], [38]. Proper function of the adenosine triphosphate (ATP)-dependent chromatin remodeling enzymes was recently found to be key for nucleosome positioning in yeast [39]–[41] and mammalian species [42]. It also suggests that transcription or the transcription initiation complexes do not play a direct role in nucleosome phasing surrounding TSSs [40], which is also supported by the fact that genes with poised Pol II in the human genome exhibited a similar pattern of nucleosome phasing to the expressed genes [13].

A barrier model was proposed to explain genome-wide nucleosome positioning [3], [43]. Nucleosomes can be organized passively at regular intervals surrounding a barrier. The barrier model can be used to explain the phased nucleosome arrays surrounding TSSs in that each TSS indirectly dictates a phased position for the next adjacent nucleosome. Whatever factors that determine spacing of nucleosomes in that context would then force the subsequent nucleosome to also be phased, and so on until an array of phased nucleosomes is formed. A barrier can only enforce its effect within a limited distance, resulting in the decay of nucleosome phasing away from the barrier. The effect of the barriers appear to be bidirectional since phased nucleosome arrays are formed on both sides of the TSSs.

Gaffney et al. (2012) recently mapped nucleosomes surrounding the binding sites of 35 different transcription factors in human lymphoblastoid cell lines. Strongly positioned nucleosome arrays were found to flank the binding sites, including those at least 1 kb away from a known TSS [10]. Phased nucleosome arrays were observed around the binding sites of other regulatory proteins, such as the mammalian insulator protein CTCF [5], [44] and repressor protein NRSF/REST [5]. Hughes et al. (2012) recently studied nucleosome positioning of *S. cerevisiae* strains containing large genomic regions from other yeast species [15]. Nucleosome-depleted regions (NDRs) fortuitously arose in coding regions of the foreign genomic sequences. Interestingly, these NDRs are associated with binding of TFIIB, an essential component of the RNA polymerase II core transcriptional machinery, and were flanked by phased nucleosomes [15]. These results are all in favor of the barrier model because the binding of a regulatory protein to both promoters and non-promoter regions can create a barrier for nucleosome organization. The regulatory proteins reported to be involved in nucleosome positioning include nucleosome remodelers and transcription factors, including activators, components of the preinitiation complex and elongating Pol II [6].

We demonstrate that DHSs in the rice genome are flanked by phased nucleosome arrays on both sides (Figure 1), which is highly similar to the nucleosome arrays flanking TSSs. Phased nucleosome arrays were associated with DHSs located in different genomic regions, including those inside of genes and intergenic regions. A similar association of DHSs with phased nucleosomes was also observed in the human genome (Figure 6). It has been well documented in different eukaryotes that DHSs represent regions associated with various regulatory proteins. For example, the binding patterns of 21 developmental regulators in *Drosophila* were quantitatively correlated with DNA accessibility in chromatin that can be measured by the DNase I sensitivity [45]. More strikingly, 94.4% of a combined 1,108,081 binding sites from all human ENCODE transcription factors fall within DHSs [23]. Similarly, we previously found that ∼90% of the binding sites of two of the best characterized transcription factors in *A. thaliana*, APETALA1 and SEPALLATA3, were covered by DHSs [46]. Thus, the association of DHSs with phased nucleosome arrays shows that the barrier model can be extended to an entire genome: any genomic region associated with regulatory proteins can serve as a barrier for nucleosome organization, and these regions can be either directly associated with transcription, such as promoters, or indirectly associated with transcription, such as the insulators. This model would also predict different nucleosome positioning profiles in different organs/tissues and in different developmental stages due to differential binding of regulatory proteins.

A DHS-based barrier can be permanent, such as the promoters associated with constitutively expressed genes, or be temporarily, such as binding sites of transcription factors associated with tissue- or organ-specific gene expression. Regulatory proteins can bind DNA tightly or loosely (or dynamically, with transient nucleosome formation in the same region), which may result in “hard” barriers or “soft” barriers. Hard barriers will result in well positioned and well phased nucleosome arrays; whereas soft barriers may result in “fuzzy” and less phased nucleosome arrays. In *Drosophila*, the binding sites of transcription factors that are flanked with strongly positioned nucleosome arrays were more sensitive to DNase I digestion and have more pronounced DNase I footprints [10]. These results support that the levels of transcription factor occupancy at the binding site determine the levels of positioning of the flanking nucleosome arrays, thus, the level of “hardness” of the barrier.

In summary, we demonstrate that DHSs located across the rice genome are flanked by strongly phased nucleosome arrays. We confirmed the same phenomenon in the human genome by analyzing publically available datasets. Our results support the barrier model for nucleosome organization as a general feature of eukaryote genomes. We propose that genome-wide nucleosome positioning in the eukaryotic genomes is orchestrated by genomic regions associated with regulatory proteins.

## Materials and Methods

### MNase-seq

Rice cultivar “Nipponbare” seeds were germinated at room temperature for three days. Germinated seeds were then sowed in soil to continue to grow in the greenhouse. The seedlings continued to grow for two weeks under 12 hrs day/night cycles and 32°C/27°C corresponding to day and night, respectively. The seedlings were then harvested for nuclei isolation, the same growing stage/condition used for developing DNase-seq and RNA-seq datasets previously [24]. The nuclei were then digested with a series of concentrations of micrococcal nuclease (MNase). The MNase-digested DNA was separated using 2% agarose gel containing ethidium bromide and visualized under UV light. Nuclei were digested into ∼80% nucleosome monomers and ∼20% dimers. The mono-nucleosomal DNA was then excised from the gel and purified using a gel purification kit (Qiagen, 28006). The purified DNA was used for MNase-seq library development, including end blunting, adding “A” base to the blunt DNA fragments, ligating “A” tailed DNA fragments with either single-end adapter or pair-end adapter, and enriching ligated DNA fragments by PCR. The final, amplified DNA was purified and sequenced with 36 bp SR (single reads) or PE (paired end) using Illumina sequencing platforms.

### Data analysis

We mapped the MNase-seq reads to the rice genome (TIGR release 5) using MAQ software [47] with default parameters (except 1-bp mismatch allowed). Only the reads aligning to a unique position in the rice genome were used for further analysis. DNase-seq and RNA-seq dataset were generated from our previous work [24]. Methods for mapping DNase-seq and RNA-seq reads were described previously [24]. We used the same methods to analyze datasets from human CD4+ T cell line, including DNase-seq dataset [32], MNase-seq dataset [13], and H2A.Z ChIP-seq [31]. All sequence reads from human CD4+ T cell line were aligned to human genome build 37 of NCBI using MAQ software using default parameters (except 1-bp mismatch allowed). We used F-seq [48] with 200-bp bandwidth parameter to identify rice DHSs. To control the FDR of the identified DHSs, we generated 10 random datasets each containing the same number of sequence reads as our DNase-seq dataset. The FDR was calculated as ratio of DHSs identified from random datasets to DHSs identified from the DNase-seq dataset. We controlled the FDR<0.05. We used the same method and parameters as Boyle et al. [32] to identify the DHSs in human CD4+ T cell line. We employed nucleR [49] to predict phased nucleosomes based on pair-end MNase-seq data using nonparametric method. We removed all fragments >200 bp (distance between the paired reads) and trimmed the fragments to the middle 40 bp to remark the position of dyad. The dyad positions were transformed by Fast Fourier Transform to show distribution of nucleosomes in Figure 3 and to identify the phased nucleosomes. The programs for data processing and statistical test were written in Perl or R (http://www.r-project.org/).

### Accession numbers

MNase-seq data has been deposited to NCBI under accession number GSE53027.

## Supporting Information

Figure S1 Patterns of nucleosome positioning around DHSs in the rice genome. The nucleosome positioning profiles were shown around the DHSs located in (A) proximal promoters (within 200 bp upstream of a TSS); (B) distal promoters (200–1000 bp upstream of a TSS); (C) within genes; (D) downstream regions of genes (within 200 bp downstream of gene transcription); (E) intergenic region and (F) 10,000 randomly selected genomic regions. Y-axes show normalized reads (read number in per bp genome in per million reads) within 1 kb upstream and downstream around the DHSs. Ellipses indicate the nucleosomes within (grey) and outside (black) of DHSs. Arrows in (a-d) indicate the direction of gene transcription. Paired MNase-seq reads were used in mapping nucleosome positioning.
(PDF)

Figure S2 An illustration of mapping phased nucleosomes with different linker lengths. After MNase digestion, linker DNA was presumably digested and the remaining DNA fragments wrapped on nucleosome core were included in library construction. Longer linkers between adjacent nucleosomes may cause a shift between the sequence reads derived from the forward and reverse strands, respectively.
(PDF)
