# Supplementary figures and images for: Genome-Wide Nucleosome Positioning Is Orchestrated by Genomic Regions Associated with DNase I Hypersensitivity in Rice
Source: PLoS Genet. 2014 May 22;10(5):e1004378. doi: 10.1371/journal.pgen.1004378 (PMC4031139; doi:10.1371/journal.pgen.1004378)

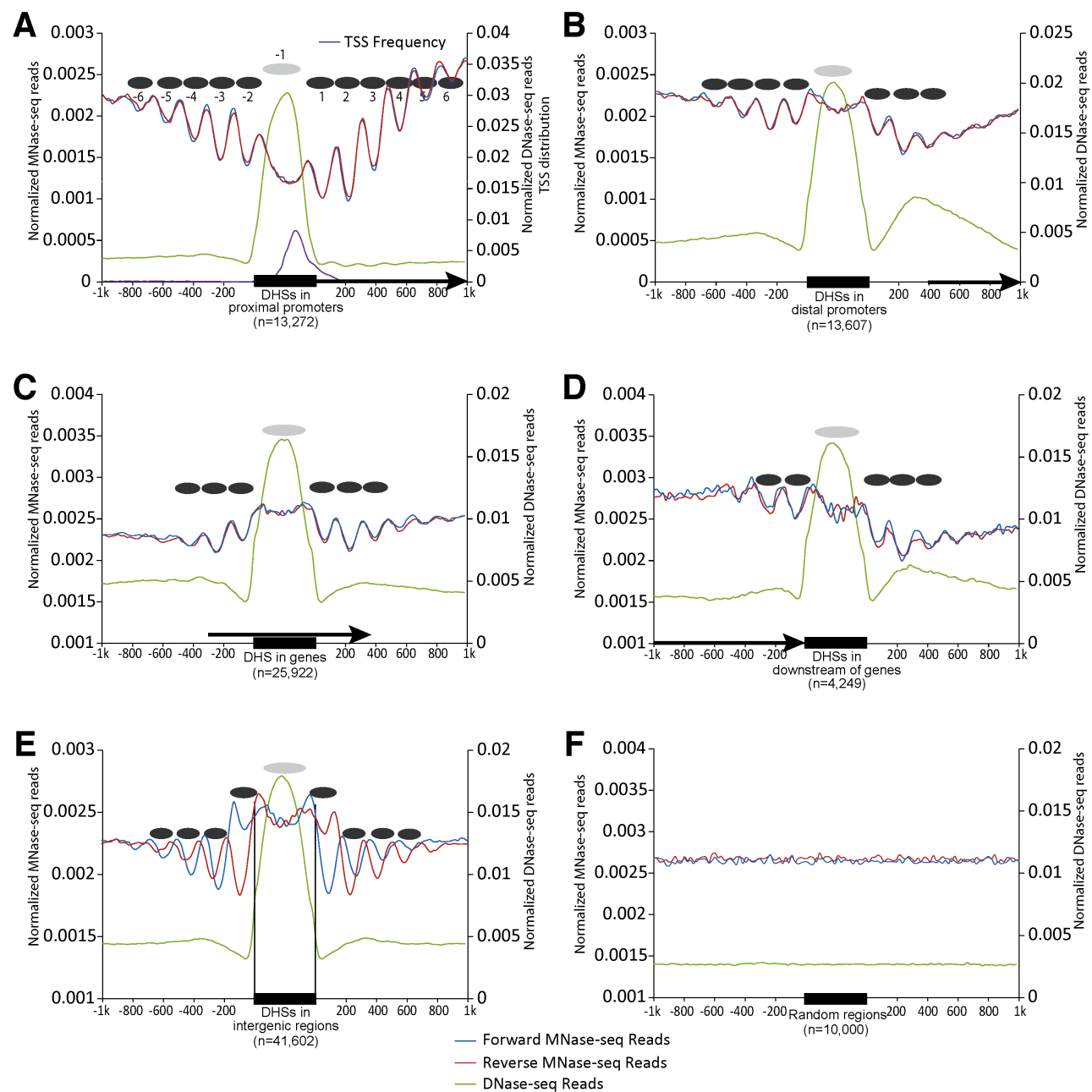

**Figure S1**

Supplement: Figure S1 — Patterns of nucleosome positioning around DHSs in the rice genome. The nucleosome positioning profiles were shown around the DHSs located in (A) proximal promoters (within 200 bp upstream of a TSS); (B) distal promoters (200–1000 bp upstream of a TSS); (C) within genes; (D) downstream regions of genes (within 200 bp downstream of gene transcription); (E) intergenic region and (F) 10,000 randomly selected genomic regions. Y-axes show normalized reads (read number in per bp genome in per million reads) within 1 kb upstream and downstream around the DHSs. Ellipses indicate the nucleosomes within (grey) and outside (black) of DHSs. Arrows in (a-d) indicate the direction of gene transcription. Paired MNase-seq reads were used in mapping nucleosome positioning. (PDF) [file pgen.1004378.s001.pdf]

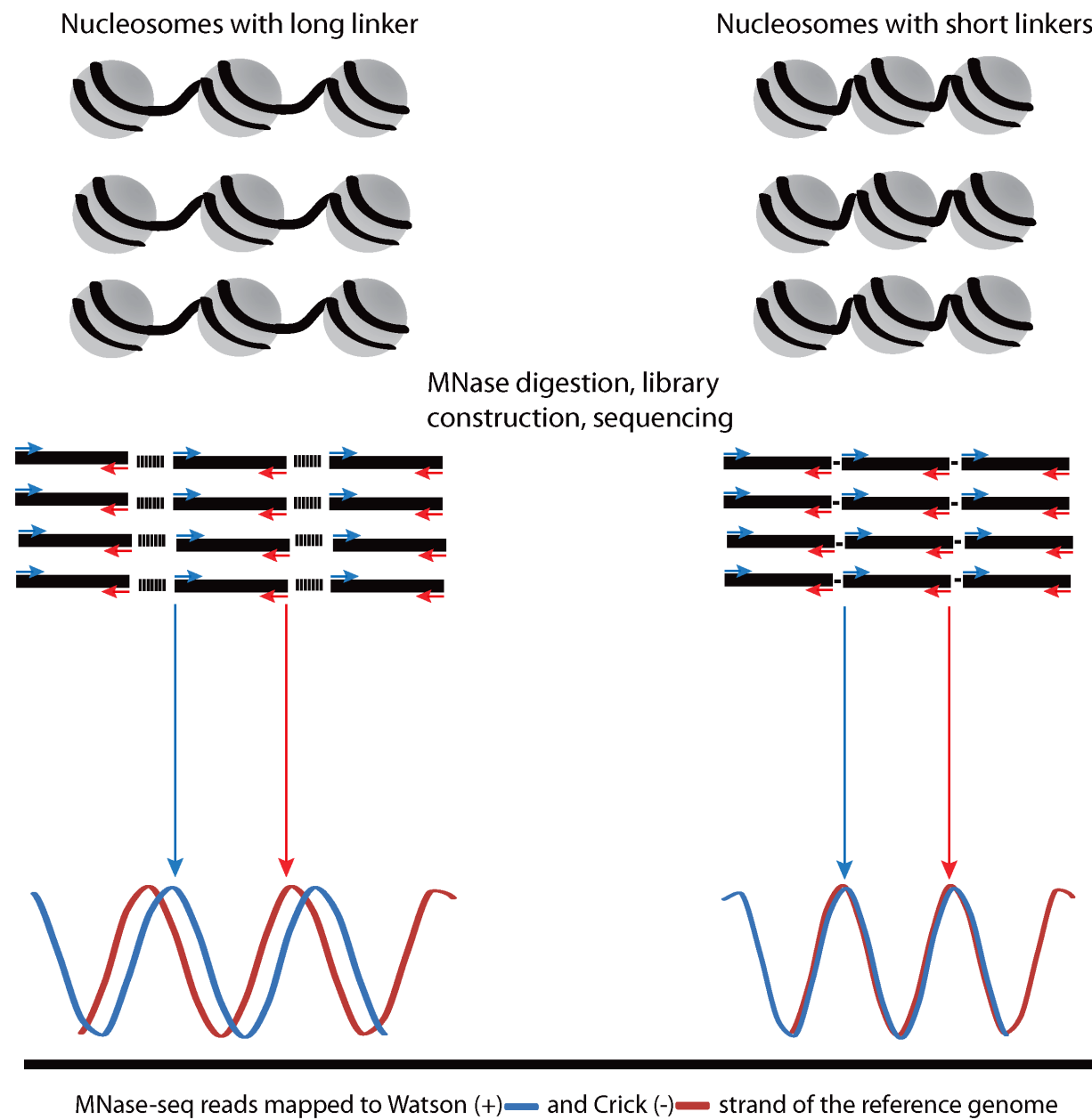

**Figure S2**

Supplement: Figure S2 — An illustration of mapping phased nucleosomes with different linker lengths. After MNase digestion, linker DNA was presumably digested and the remaining DNA fragments wrapped on nucleosome core were included in library construction. Longer linkers between adjacent nucleosomes may cause a shift between the sequence reads derived from the forward and reverse strands, respectively. (PDF) [file pgen.1004378.s002.pdf]
